# Cannabinoid Receptor Type 1 in Parkinson's Disease: A Positron Emission Tomography Study with [ ^18^F]FMPEP‐*d*_2_

**DOI:** 10.1002/mds.29117

**Published:** 2022-06-08

**Authors:** Riikka M. Ajalin, Haidar Al‐Abdulrasul, Jouni M. Tuisku, Jussi E.S. Hirvonen, Tero Vahlberg, Salla Lahdenpohja, Juha O. Rinne, Anna E. Brück

**Affiliations:** ^1^ Turku PET Centre, Turku University and Turku University Hospital Turku Finland; ^2^ Neurocenter, Turku University Hospital and Clinical Neurosciences University of Turku Turku Finland; ^3^ Department of Neurology, Helsinki University Hospital and Department of Clinical Neurosciences (Neurology) University of Helsinki Helsinki Finland; ^4^ Department of Radiology University of Turku and Turku University Hospital Turku Finland; ^5^ Department of Biostatistics University of Turku Turku Finland

**Keywords:** Parkinson's disease, PET, CB1 receptor, dopaminergic medication, endocannabinoid system

## Abstract

**Background:**

The endocannabinoid system is a widespread neuromodulatory system affecting several biological functions and processes. High densities of type 1 cannabinoid (CB1) receptors and endocannabinoids are found in basal ganglia, which makes them an interesting target group for drug development in basal ganglia disorders such as Parkinson's disease (PD).

**Objective:**

The aim of this study was to investigate CB1 receptors in PD with [^18^F]FMPEP‐*d*_2_ positron emission tomography (PET) and the effect of dopaminergic medication on the [^18^F]FMPEP‐*d*_2_ binding.

**Methods:**

The data consisted of 16 subjects with PD and 10 healthy control subjects (HCs). All participants underwent a [^18^F]FMPEP‐*d*_2_ high‐resolution research tomograph PET examination for the quantitative assessment of cerebral binding to CB1 receptors. To investigate the effect of dopaminergic medication on the [^18^F]FMPEP‐*d*_2_ binding, 15 subjects with PD underwent [^18^F]FMPEP‐*d*_2_ PET twice, both *on* and *off* antiparkinsonian medication.

**Results:**

[^18^F]FMPEP‐*d*_2_ distribution volume was significantly lower in the *off* scan compared with the *on* scan in basal ganglia, thalamus, hippocampus, and amygdala (*P* < 0.05). Distribution volume was lower in subjects with PD *off* than in HCs globally (*P* < 0.05), but not higher than in HCs in any brain region.

**Conclusions:**

Subjects with PD have lower CB1 receptor availability compared with HCs. PD medication increases CB1 receptor toward normal levels. © 2022 The Authors. *Movement Disorders* published by Wiley Periodicals LLC on behalf of International Parkinson and Movement Disorder Society

## Graphical abstract

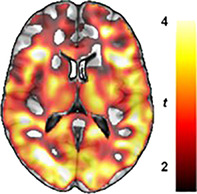

The endocannabinoid system (ECS) is a widespread and complex neuromodulatory and homeostatic system affecting several biological functions and processes, such as development of the central nervous system,^1^ cognitive processes,^2^ regulation of emotions and responses to endogenous and environmental insults,^3^ intestinal and metabolic functions,^4^, ^5^ and reproduction.^6^ The ECS includes cannabinoid receptor types 1 and 2 (CB1 and CB2), two endogenous receptor ligands or endocannabinoids, and metabolic enzymes.^7^ CB1 is most abundantly expressed in the brain and is primarily presynaptic and inhibits GABAergic and glutamatergic transmission.^7^, ^8^, ^9^

High densities of CB1 receptors and endocannabinoids are found in basal ganglia,^10^, ^11^, ^12^ which makes them an interesting target group for drug development in basal ganglia disorders such as Parkinson's disease (PD). Experimental PD models and postmortem PD studies have shown CB1 receptors to be downregulated in the presymptomatic stages of PD, which in turn is believed to lead to increased oxidative stress and disease progression through the enhanced glutamate levels and excitotoxicity.^13^, ^14^ As the disease progresses, a significant upregulation of CB1 receptors is seen as an adaptive response to the changing environment.^14^, ^15^

A positron emission tomography (PET) study using CB1 receptor selective tracer [^18^F]MK9470 showed subjects with PD to have a reduction in CB1 availability in substantia nigra compared with healthy control subjects (HCs), and a relative increase of CB1 in nigrostriatal, mesolimbic, and mesocortical pathways and in the putamen contralateral to the most affected body side of subjects with PD.^16^ Later it was shown that decreased [^18^F]MK9470 binding in the prefrontal and midcingulate cortex in subjects with PD correlated with disturbances in executive functioning, episodic memory, and visuospatial functioning.^17^ However, these two studies are the only in vivo studies investigating the ECS in subjects with idiopathic PD. Furthermore, the investigations were performed only at voxel level, and the PET imaging was done *on* medication.

[^18^F]FMPEP‐*d*_2_ is an inverse agonist radioligand that has high affinity and selectivity for the CB1 receptor and has a markedly smaller intersubject variability than [^18^F]MK9470. Also, compared with other CB1 receptor ligands, it seems to provide the most accurate measurements.^18^, ^19^, ^20^ So far it has been used clinically in studies on cannabis smokers,^21^ subjects with alcohol dependency,^22^ tobacco smokers,^23^ subjects with first‐episode psychosis,^24^ and healthy volunteers.^25^, ^26^

The aim of this study was to compare [^18^F]FMPEP‐*d*_2_ binding between subjects with PD and HCs, both at volume of interest and voxel level. In addition, we also examined whether the dopaminergic medication had an effect on [^18^F]FMPEP‐*d*_2_ binding in subjects with PD.

## Subjects and Methods

### Subjects and Study Design

The study was approved by the Ethics Committee of the Hospital District of Southwest Finland and was conducted according to the World Medical Association Declaration of Helsinki (Ethical Principles for Medical Research Involving Human Subjects) and following Good Clinical Practice guidelines. All participants gave their written informed consent obtained according to the Declaration of Helsinki.

The study sample consisted of 16 subjects with PD and 10 HCs (Table 1). All participants were interviewed thoroughly for their medical history. A neurological examination was performed by a clinical neurologist. Participants with a significant neurological illness other than PD, major psychiatric illness such as schizophrenia, a clinical history of stroke or evidence of focal brain lesions on magnetic resonance imaging (MRI), contraindication for an MRI, or previous participation in research studies involving significant radiation exposure were excluded. The PD and HC groups did not differ in age, sex, body mass index, or injected tracer activity.

**TABLE 1:** Demographics and characteristics: demographic information of groups (top) and clinical information of subjects with Parkinson's disease (bottom)

	PD *On*	PD *Off*	HC	*P* Value
No. of subjects (male/female)	9/6	9/7	5/5	–
Age (y)	65 ± 6	66 ± 6	67 ± 8	0.968
Body mass index (kg/m^2^)	29 ± 6	29 ± 6	25 ± 5	0.148
Injected activity of [^18^F]FMPEP‐*d*_2_ (MBq)	195 ± 12	201 ± 12	200 ± 15	0.477
Smoking of any kind	–	–	–	–
Hormone replacement therapy	–	–	–	–
	Mean ± SD	Minimum	Maximum	
UPDRS‐III (*off* medication)	22 ± 9	8	36	
LEDD (mg)	546 ± 195	207	1007	
Disease duration (y)	9 ± 6	2	19	

Values are number (n) or mean ± SD.

PD, Parkinson's disease; HC, healthy control subject; UPDRS‐III, Unified Parkinson's Disease Rating Scale‐motor, Part III; LEDD, levodopa equivalent daily dose; SD, standard deviation.

Recruited participants with PD fulfilled the UK Brain Bank Research criteria for the diagnosis of idiopathic PD. To confirm the clinical diagnosis of PD, all PD participants also underwent [^18^F]FDOPA high‐resolution research tomograph PET examination showing typical nigrostriatal dopaminergic hypofunction for PD. All subjects with PD were on their individual standard PD medication, including dopamine agonists (15 of the subjects), levodopa,^15^ and monoamine oxidase B inhibitors.^13^ The details on the dopaminergic medication for each subject are presented in Supporting Information Table S1. None of the participants were using hormone replacement therapy or reported smoking of any kind.

### Radiochemistry

[^18^F]FMPEP‐*d*_2_ was prepared as described previously.^27^ The radioligand was obtained in high radiochemical purity (>95%) and had a molar activity of 200 ± 15 MBq/nmol at the time of injection.

### Imaging Procedures

Structural MRI was performed with a 3‐T scanner (Philips Ingenuity TF PET/MR; Philips Medical Systems, Cleveland, OH, USA) and evaluated by an experienced clinical neuroradiologist for the exclusion of contributing pathologies. Three‐dimensional T1‐weighted MRIs were used for delineation of anatomical regions after coregistration with PET data.

The [^18^F]FMPEP‐*d*_2_ examinations were performed with a high‐resolution research tomograph PET scanner (Siemens/CTI, Knoxville, TN, USA). An individually molded thermoplastic mask with a head fixation system was used to minimize head movement during the scan. After a 6‐minute transmission scan (using ^137^Cs point source), [^18^F]FMPEP‐*d*_2_ was injected intravenously as a rapid bolus and followed by 60‐minute dynamic PET acquisition. This was followed by a 30‐minute pause, during which participants were allowed to come out from the scanner for comfort reasons. The scan was then continued for another 30 minutes for a total emission data scan range of 0–60 and 90–120 minutes. After the scan, another 6‐minute transmission scan was performed.

During the first 3.5 minutes of the scanning, continuous blood sampling was performed by an automated blood sampling system (Allogg ABSS; Allogg AB, Mariefred, Sweden, https://www.allogg.se). After that, blood samples were drawn manually from a radial artery cannula at different time points as described previously.^25^ On these time points, blood plasma radioactivity was measured, and the metabolites of [^18^F]FMPEP‐*d*_2_ were determined to generate the time curve function for plasma radioactivity of unchanged radioligand. Hill‐type function was fitted to individual parent fraction measurements, after which the metabolite‐corrected plasma time‐activity curves were calculated by multiplying the uncorrected plasma curves with the Hill model curves. This represents the input function in the later analyses.

To investigate the dopaminergic drug effect, 15 PD subjects underwent two [^18^F]FMPEP‐*d*_2_ PET examinations. Levodopa medication was discontinued 12 hours and other dopaminergic medication 24 hours before the *off*‐medication scan (PD *off*). Participants could continue their medication immediately after the scan. Another [^18^F]FMPEP‐*d*_2_ examination was performed on a different day without drug interruption (PD *on*). There was an average of 101 days (minimum 7 days, maximum 428 days) between the scans. In addition, one PD subject underwent only one [^18^F]FMPEP‐*d*_2_ examination with the interruption of medication.

### PET Imaging Analyses

The PET data were realigned and coregistered with anatomical T1‐weighted MRIs using SPM12 software (Wellcome Trust Centre for Neuroimaging, London, UK) running in MATLAB (The MathWorks, Natick, MA, USA). Fourteen bilateral regions of interest (ROIs; putamen, caudate nucleus, substantia nigra, globus pallidus, thalamus, amygdala, hippocampus, insula, cingulate cortex, cerebellum, and frontal, parietal, temporal, and occipital lobes) were generated with the FreeSurfer software (version 6).^28^ MNI152 template‐based anatomical substantia nigra ROI located in the ventral midbrain^29^ (https://identifiers.org/neurovault.collection:2860) was first mapped to subject native space with the deformation field obtained from SPM segmentation, after which the individual ROIs were manually checked by a neurologist.

For quantitative assessment of [^18^F]FMPEP‐*d*_2_ binding, regional and voxel‐level distribution volumes (*V*_T_s) were estimated using Logan's method within 30–120 minutes, where the plasma time‐activity curves were set as an input function. Logan plot has been shown to be highly correlated with two‐tissue compartmental modeling.^30^ To reduce the effect of noise, we smoothed the PET data with Gaussian 2‐mm full width at half maximum filter before the voxel‐level modeling.

### Statistical Analyses

Statistical analyses were performed with IBM SPSS Statistics program (version 26). Shapiro‐Wilks's test and visual inspection of Q–Q plots was used to test the normality assumption. *V*_T_ values were normally distributed except for 2 of 28 brain regions in the PD *on* group, for 1 brain region in the PD *off* group, and for 4 brain regions in HCs. Variance was homogeneous across groups according to Levene's test. Baseline characteristics of participants (Table 1) were compared using two‐sample *t* tests. Two‐sample *t* test was also used to compare *V*_T_ values between sexes. Results were confirmed with nonparametric Mann–Whitney *U* test when normality assumption was violated. Correlations between brain regions and age and disease duration were assessed with Pearson's *r* correlations and Spearman's rho when normality assumption was violated.

To compare with and without medication interruption examinations (PD *off* vs. *on*), we used paired‐samples *t* test to analyze [^18^F]FMPEP‐*d*_2_ *V*_T_s among the 15 PD subjects who had both scans. Related‐samples Wilcoxon signed rank test was used to confirm the results on those brain regions where normal distribution assumption was violated. Paired‐samples *t* test and related‐samples Wilcoxon signed rank test were also used to compare *V*_T_ values between hemispheres. The differences in the regional *V*_T_ between groups (PD vs. HC) were analyzed using general linear model with group status and sex as fixed factors, age as a covariate, and brain regions as dependent variables. The results were confirmed with nonparametric Mann–Whitney *U* tests when normality assumption was violated. The level of significance was set as *P* < 0.05 in all tests.

### Voxel‐Wise Analysis of *V*_T_

To confirm results from volume‐of‐interest analysis and to explore regional specificity of findings, we compared parametric *V*_T_ maps between groups at voxel level using SPM12. Before the analysis, *V*_T_ maps were normalized to MNI512 space using clinical toolbox^31^ in SPM12 and spatially smoothed using Gaussian 8‐mm full width at half maximum filter. A two‐sample *t* test was done to compare PD and HC groups, with age and sex as covariates. A paired‐samples *t* test was done to compare PD *off* and *on* groups to investigate the effect of dopaminergic medication to [^18^F]FMPEP‐*d*_2_ binding. All voxel‐level results were corrected for multiple comparisons by using false discovery rate at *P* < 0.05.

## Results

There were no significant correlations between age and *V*_T_ of [^18^F]FMPEP‐*d*_2_ in any group. There were also no significant correlations between disease duration and *V*_T_ of [^18^F]FMPEP‐*d*_2_ in PD groups. Furthermore, no significant differences in [^18^F]FMPEP‐*d*_2_ *V*_T_ were observed between males and females in the PD groups. In the HC group, [^18^F]FMPEP‐*d*_2_ *V*_T_ was significantly higher in females compared with males in five brain areas (mean ± standard deviation): left occipital lobe (females: 11.0 ± 2.1; males: 7.8 ± 0.7; *P* = 0.012), right occipital lobe (females: 11.4 ± 2.4; males: 7.9 ± 0.8; *P* = 0.014), left parietal lobe (females: 13.8 ± 2.3; males: 10.5 ± 1.2; *P* = 0.021), right parietal lobe (females: 13.8 ± 2.1; males: 10.6 ± 1.2; *P* = 0.02), and left cerebellum (females: 9.5 ± 1.8; males: 7.2 ± 1.0; *P* = 0.035).

### [ ^18^F]FMPEP‐*d*_2_ Binding in PD and HC Groups

When comparing the PD *off* group with the HC group, lower [^18^F]FMPEP‐*d*_2_ *V*_T_ was seen in all brain regions, with percent decrease ranging from 15% to 28% on the left side and from 7% to 22% on the right side. Statistically significantly lower *V*_T_ was seen bilaterally in caudate nucleus (left: *P* = 0.028; right: *P* = 0.040), thalamus (left: *P* = 0.007; right: *P* = 0.020), and parietal (left: *P* = 0.011; right: *P* = 0.026) and occipital lobes (left: *P* = 0.004; right: *P* = 0.011). In addition, lower *V*_T_ was seen unilaterally in substantia nigra (*P* = 0.011), hippocampus (*P* = 0.030), insula (*P* = 0.039), and temporal lobe (*P* = 0.016) on the left side. For the *V*_T_ values, mean difference, and *P* values of every studied region, see Table 2. When comparing the PD *on* group with the HC group, a statistically significantly lower *V*_T_ was seen in occipital lobes (left: *P* = 0.019; right: *P* = 0.043) and left substantia nigra (*P* = 0.015). A similar trend was seen in all brain regions.

**TABLE 2:** [^18^F]FMPEP‐*d*_2_ binding in Parkinson's disease *off* and healthy control groupsBrain Region

	Left Hemisphere					Right Hemisphere				
	*V*_T_ *off*, mL/cm^3^	*V*_T_ HC, mL/cm^3^	Mean Difference (CI), mL/cm^3^	*P* Value	Percentage Difference, *Off* < HC (%)	*V*_T_ *Off*, mL/cm^3^	*V*_T_ HC, mL/cm^3^	Mean Difference (CI), mL/cm^3^	*P* Value	Percentage Difference, *off* < HC (%)
FRO	10.6 ± 2.3	12.7 ± 2.2	−2.11 (−3.98, −0.24)	0.053^a^	17	11.0 ± 2.4	12.8 ± 2.2	−1.83 (−3.77, 0.10)	0.060	14
PAR	9.8 ± 2.1	12.1 ± 2.5	−2.35 (−4.21, −0.49)	0.011*	19	10.1 ± 2.2	12.2 ± 2.3	−2.07 (−3.97, −0.18)	0.026*	17
TMP	10.3 ± 2.4	12.9 ± 2.5	−2.59 (−4.63, −0.56)	0.016*	20	12.2 ± 6.4	13.2 ± 2.6	−1.00 (−5.44, −3.43)	0.087^a^	8
OCC	7.2 ± 1.7	9.4 ± 2.3	−2.18 (−3.78, −0.59)	0.004*	23	7.6 ± 1.7	9.6 ± 2.5	−2.02 (−3.73, −0.32)	0.011*	21
CIN	10.8 ± 2.5	12.8 ± 2.3	−1.97 (−3.98, 0.04)	0.054	15	10.8 ± 2.6	12.6 ± 2.6	−1.76 (−3.92, 0.41)	0.115	14
CAU	8.0 ± 2.0	9.9 ± 2.0	−1.88 (−3.55, −0.22)	0.028*	19	8.3 ± 1.9	10.2 ± 2.6	−1.87 (−3.70, −0.03)	0.040*	18
PUT	11.5 ± 2.9	13.8 ± 2.9	−2.23 (−4.64, 0.18)	0.077	16	11.7 ± 2.6	13.6 ± 3.2	−1.91 (−4.26, 0.44)	0.107	14
THA	4.8 ± 1.3	6.5 ± 1.4	−1.63 (−2.72, −0.54)	0.007*	25	5.0 ± 1.4	6.4 ± 1.2	−1.38 (−2.46, −0.29)	0.020*	22
HIP	8.3 ± 2.2	10.7 ± 2.9	−2.43 (−4.50, −0.35)	0.030*	23	8.8 ± 2.4	10.6 ± 3.1	−1.76 (−3.96, 0.45)	0.117	17
AMG	9.9 ± 3.9	12.4 ± 3.3	−2.51 (−5.58, 0.55)	0.142	20	9.7 ± 3.6	12.3 ± 4.4	−2.66 (−5.93, 0.60)	0.110	22
INS	11.1 ± 2.8	13.7 ± 2.7	−2.55 (−4.84, −0.26)	0.039*	19	11.1 ± 2.9	13.1 ± 2.9	−2.10 (−4.48, 0.29)	0.075	16
PAL	10.8 ± 3.7	13.3 ± 2.7	−2.52 (−5.29, 0.24)	0.094	19	11.3 ± 3.6	13.6 ± 3.7	−2.28 (−5.29, 0.72)	0.135	17
CER	7.1 ± 2.0	8.4 ± 1.9	−1.29 (−2.89, 0.30)	0.097	15	7.2 ± 2.0	8.5 ± 1.9	−1.33 (−2.98, 0.32)	0.101	16
SN	5.9 ± 1.8	8.2 ± 2.4	−2.27 (−3.99, −0.54)	0.011*	28	6.9 ± 3.7	7.4 ± 2.6	−0.53 (−3.32, 2.26)	0.286^a^	7

Values are mean ± SD from the age‐ and sex‐adjusted general linear model.

* *P* < 0.05.

^a^ Nonparametric Mann–Whitney *U* test when normality assumption was violated.

*V*_T_, distribution volume; HC, healthy control; CI, 95% confidence interval; FRO, frontal lobe; PAR, parietal lobe; TMP, temporal lobe; OCC, occipital lobe; CIN, cingulate cortex; CAU, caudate nucleus; PUT, putamen; THA, thalamus; HIP, hippocampus; AMG, amygdala; INS, insula; PAL, globus pallidus; CER, cerebellum; SN, substantia nigra; SD, standard deviation.

**FIG 1:**
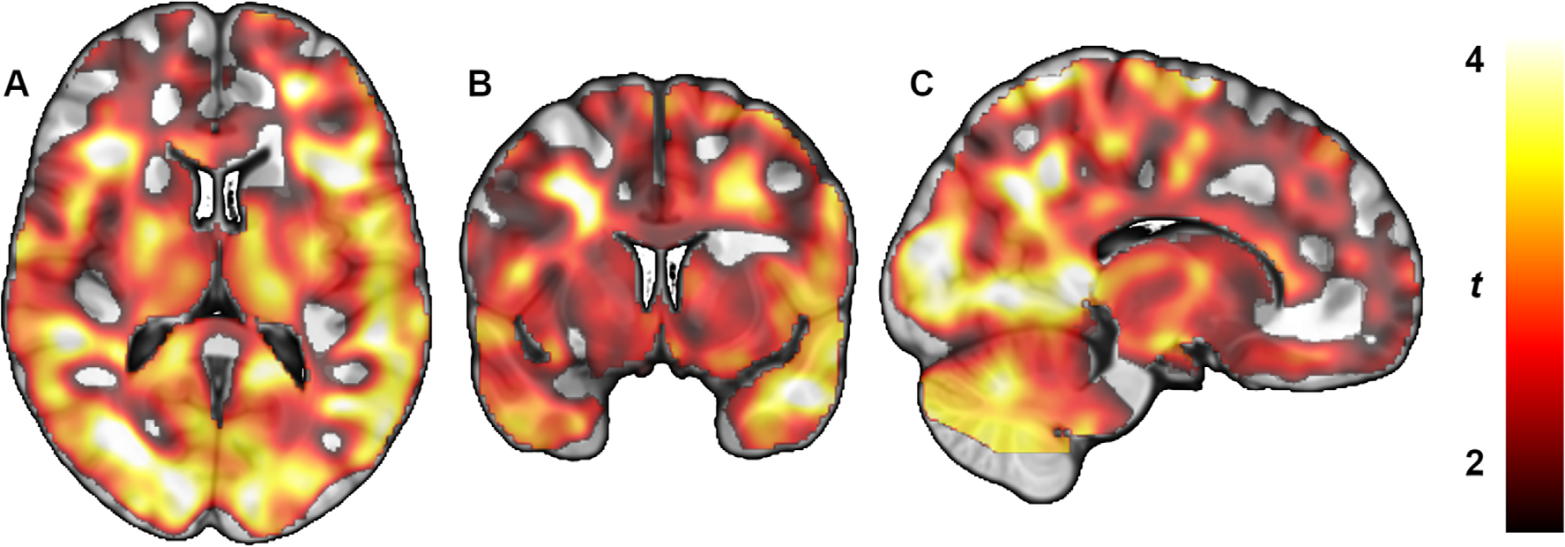
Whole‐brain statistical parametric mapping analysis shows lower type 1 cannabinoid receptor density (distribution volume) in subjects with Parkinson's disease compared with healthy control subjects. Color bar represents *t* value, which corresponds to the level of significance at the voxel level. (**A**) Transaxial section. (**B**) Coronal section. (**C**) Sagittal section.

In the voxel‐wise analysis, similar group differences were observed between the PD *off* and HC groups. The PD *off* subjects showed statistically significant clusters of lower *V*_T_ compared with HCs. The clusters of lower binding were located widespread over both hemispheres. Local maxima were observed in the middle, inferior, and superior temporal gyri, associative visual cortex, and angular gyrus (Fig. 1). The PD *on* subjects showed less and markedly smaller clusters of lower *V*_T_ compared with HCs. The clusters included bilateral parts of the dorsal and ventral posterior cingulate cortices and secondary and associative visual cortices. No region showed higher [^18^F]FMPEP‐*d*_2_ binding in PD groups compared with HCs.

No significant main effect of age was seen in any studied brain region. The main effect of sex was significant only in occipital lobes (right: *P* = 0.037; left: *P* = 0.023), where females had higher levels (right: 9.3 ± 2.8; left: 9.0 ± 2.4) compared with males (right: 7.6 ± 1.7; left: 7.3 ± 1.6) and similarly in right amygdala (females: 12.5 ± 4.6; males: 9.1 ± 2.9; *P* = 0.03). There were no significant differences between hemispheres in the HC group. In the PD *off* group, the only significant difference between hemispheres was seen in the occipital lobe, where the right side had statistically higher *V*_T_ compared with the left side (*P* = 0.006).

### Effect of Dopaminergic Medication on [ ^18^F]FMPEP‐*d*_2_ Binding

**FIG 2:**
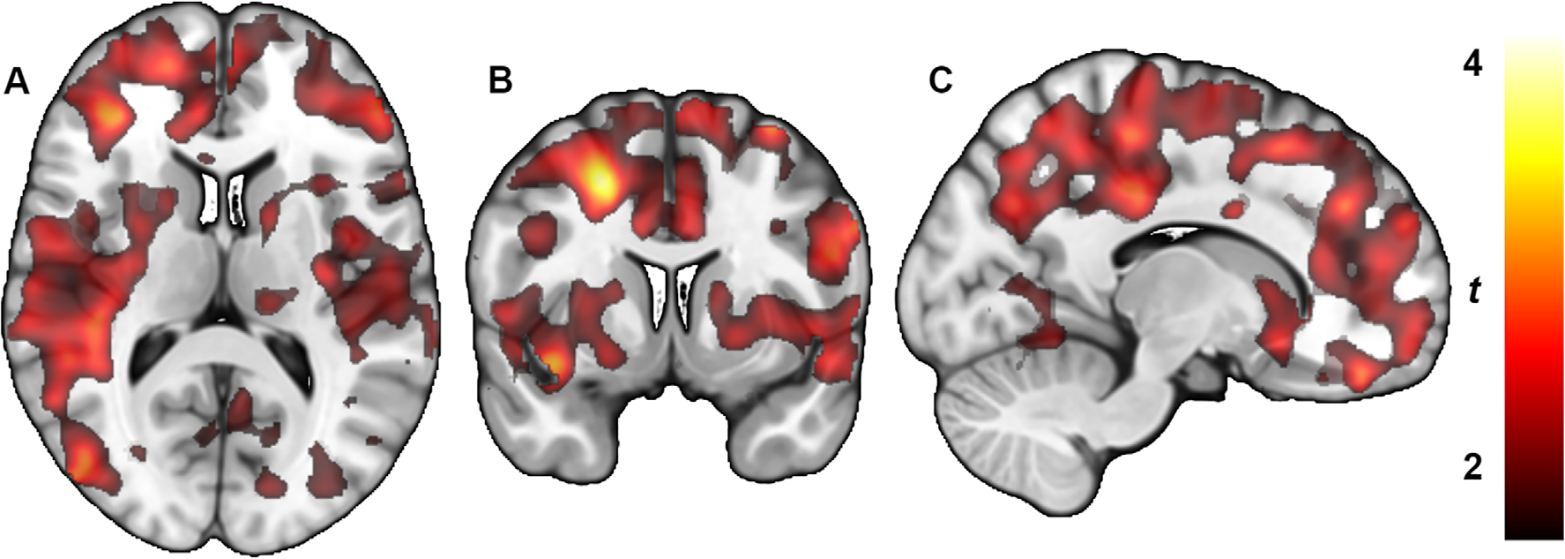
Whole‐brain statistical parametric mapping analysis shows lower type 1 cannabinoid receptor density (distribution density) in subjects with Parkinson's disease when they were *off* antiparkinsonian medication compared with being *on* their usual medication. Color bar represents *t* value, which corresponds to the level of significance at the voxel level. (**A**) Transaxial section. (**B**) Coronal section. (**C**) Sagittal section.

[^18^F]FMPEP‐*d*_2_ *V*_T_ was significantly lower in the *off* scan compared with the *on* scan bilaterally in the caudate nucleus (left: *P* = 0.004; right: *P* = 0.005). Unilaterally [^18^F]FMPEP‐*d*_2_ *V*_T_ was significantly lower in the *off* scan compared with the *on* scan on the left side in thalamus (*P* = 0.007), hippocampus (*P* = 0.024), amygdala (*P* = 0.008), and globus pallidus (*P* = 0.031) and on the right side in putamen (*P* = 0.026). A similar trend was also observed in other studied regions. See Table 3 for the *V*_T_ and *P* values of every region studied. Voxel‐wise analysis resulted in significant clusters, which were located widespread over both hemispheres (Fig. 2). In addition, the analyses were performed according to the ipsilateral and contralateral to the most affected body side. The results are very similar to the analyses performed according to the right and left sides.

**TABLE 3:** The effect of dopaminergic medication on [^18^F]FMPEP‐*d*_2_ binding

Brain Region	Left Hemisphere					Right Hemisphere				
	*V*_T_ *On*, mL/cm^3^	*V*_T_ *Off*, mL/cm^3^	Mean Difference (CI), mL/cm^3^	*P* Value	Percentage Difference, *Off* < *On* (%)	*V*_T_ *On*, mL/cm^3^	*V*_T_ *Off*, mL/cm^3^	Mean Difference (CI), mL/cm^3^	*P* Value	Percentage Difference, *Off* < *On* (%)
FRO	11.4 ± 2.8	10.5 ± 2.3	−0.93 (−2.22, 0.37)	0.147	8	11.8 ± 2.7	10.9 ± 2.4	−0.95 (−2.33, 0.42)	0.158	8
PAR	10.5 ± 2.5	9.7 ± 2.1	−0.84 (−1.89, 0.21)	0.110	8	10.9 ± 2.3	10.0 ± 2.2	−0.95 (−2.24, 0.35)	0.138	9
TMP	11.1 ± 3.0	10.2 ± 2.5	−0.82 (−2.13, 0.48)	0.198	7	12.1 ± 2.8	12.1 ± 6.6	0.02 (−3.25, 3.28)	0.140^a^	0
OCC	7.7 ± 1.7	7.1 ± 1.7	−0.56 (−1.40, 0.28)	0.173	7	8.2 ± 1.7	7.5 ± 1.8	−0.65 (−1.52, 0.22)	0.130	8
CIN	11.8 ± 3.1	10.6 ± 2.4	−1.22 (−2.57, 0.13)	0.073	10	11.8 ± 3.2	10.6 ± 2.5	−1.20 (−2.47, 0.06)	0.061	10
CAU	9.4 ± 2.8	8.0 ± 2.1	−1.41 (−2.28, −0.53)	0.004*	15	9.7 ± 2.5	8.3 ± 2.0	−1.36 (−2.24, −0.48)	0.005*	14
PUT	12.6 ± 3.5	11.4 ± 2.9	−1.20 (−2.63, 0.22)	0.092	10	12.9 ± 3.0	11.6 ± 2.6	−1.39 (−2.58, −0.19)	0.026*	11
THA	5.4 ± 1.4	4.8 ± 1.3	−0.59 (−0.99, −0.19)	0.007*	11	5.5 ± 1.4	5.0 ± 1.4	−0.52 (−1.04, 0.01)	0.052	9
HIP	9.8 ± 3.6	8.2 ± 2.2	−1.61 (−2.99, −0.24)	0.024*	16	10.0 ± 3.2	8.6 ± 2.3	−1.33 (−2.73, 0.07)	0.061	13
AMG	12.2 ± 5.3	9.2 ± 2.9	−2.96 (−5.02, −0.89)	0.008*	24	10.4 ± 5.2	9.2 ± 3.2	−1.18 (−4.69, 2.34)	0.484	11
INS	11.9 ± 3.4	11.0 ± 2.8	−0.91 (−2.49, 0.68)	0.241	8	12.1 ± 3.2	10.9 ± 2.9	−1.21 (−2.56, 0.13)	0.073	10
PAL	14.3 ± 10.9	10.4 ± 3.4	−3.95 (−9.15, 1.24)	0.031^a^ ^,^ *	28	12.3 ± 5.9	11.2 ± 3.6	−1.12 (−4.15, 1.91)	0.441	9
CER	7.9 ± 2.5	7.1 ± 2.0	−0.81 (−1.80, 0.19)	0.103	10	7.9 ± 2.4	7.2 ± 2.1	−0.73 (−1.58, 0.13)	0.090	9
SN	6.0 ± 1.6	5.9 ± 1.9	−0.16 (−1.17, 0.85)	0.739	3	6.0 ± 2.2	6.7 ± 3.8	0.74 (−1.16, 2.64)	0.570^a^	−12

Values are mean ± SD from paired‐sample *t* test.

* *P* < 0.05.

^a^ Related‐samples Wilcoxon signed rank test when normality assumption was violated.

*V*_T_, distribution volume; *On*, *on* medication; *Off*, *off* medication; CI, 95% confidence interval; FRO, frontal lobe; PAR, parietal lobe; TMP, temporal lobe; OCC, occipital lobe; CIN, cingulate cortex; CAU, caudate nucleus; PUT, putamen; THA, thalamus; HIP, hippocampus; AMG, amygdala; INS, insula; PAL, globus pallidus; CER, cerebellum; SN, substantia nigra.

## Discussion

In this study, we found that subjects with PD, who had discontinued their usual antiparkinsonian medication at least 12 hours before scanning, had significantly lower [^18^F]FMPEP‐*d*_2_ binding compared with HCs in several brain regions. In addition, when the subjects with PD were on their usual antiparkinsonian medication, the [^18^F]FMPEP‐*d*_2_ binding was significantly increased toward the normal level, indicating that antiparkinsonian medication affects the [^18^F]FMPEP‐*d*_2_ binding.

Our findings support the previous observations within the study using [^18^F]MK9470,^16^ although we did see more brain regions along with substantia nigra presenting an absolute decrease of CB1 availability. In neither study did the PD group show increased CB1 availability compared with the controls. In the [^18^F]MK9470 study, they had three different types of PD subject: a group with drug‐naive early PD subjects (disease duration 2.1 ± 1.4 years) and two groups of advanced PD subjects, with and without levodopa‐induced dyskinesia (disease durations of 12.2 ± 4.3 and 11.2 ± 3.4 years). In the [^18^F]MK9470 study, the PET imaging was done *on* medication in the advanced PD groups, which might explain why they saw decreased CB1 availability only in substantia nigra, as we observed that the antiparkinsonian medication increases the CB1 availability in subjects with PD.

The demographic factors should be noted also because previous studies have indicated a biphasic dysregulation of CB1 receptors. It has been suggested that the CB1 receptors are downregulated in early PD and upregulated in the later stages of the disease.^14^, ^15^ In our study, the disease duration did not correlate to the *V*_T_ of [^18^F]FMPEP‐*d*_2_ in PD groups. The disease duration of the PD subjects in our study was 9 ± 6 years, which is similar to the advanced PD groups in the [^18^F]MK9470 study. In the [^18^F]MK9470 study, all three PD groups showed the decreased CB1 availability in substantia nigra. In addition, with [^18^F]MK9470, a relative increase of CB1 availability in nigrostriatal, mesolimbic, and mesocortical dopaminergic projection areas was observed when [^18^F]MK9470 availability was scaled relative to the global mean per subject. These relative findings were somewhat widespread in the advanced PD group compared with the early PD group. In this study, no relative differences were studied.

In this study, we observed significantly decreased [^18^F]FMPEP‐*d*_2_ binding in subjects with PD when they were *off* antiparkinsonian medication compared with being *on* medication in the putamen, thalamus, amygdala, and hippocampus. There was an average of 101 days between the scans. Only two of the subjects had more than 5 months between the scans (251 and 428 days), but it was ensured that these subjects were not outliers, and the results remained the same when these two were excluded. It is highly unlikely that disease progression in such a short time would explain the decreased [^18^F]FMPEP‐*d*_2_ binding observed in this study. Also, when the subjects with the largest levodopa equivalent daily dose and with the longest disease duration were excluded, the results remained the same.

The functional interaction between the ECS and the dopaminergic system has previously been observed in animal studies and human studies. In a rat model of PD, increased endocannabinoid anandamide levels and decreased activity of its membrane transporter and hydrolase (fatty acid amide hydrolase) were normalized with chronic levodopa treatment.^32^ A similar finding was later reported in a human study as elevated CSF anandamide levels in untreated subjects with PD were normalized with chronic levodopa treatment.^33^ The effect of dopaminergic medication on the ECS was also demonstrated in this study as the change in CB1 receptor availability was elevated closer to normal when the subjects were on their usual dopaminergic medication.

We believe that changes in *V*_T_ between the *on* and *off* conditions reflect changes in CB1 receptor availability, rather than a methodological confound related to the dopaminergic drug treatment. Such treatment might change the peripheral metabolism of pharmacokinetics of the radioligand, but the outcome measure *V*_T_ corrects for such changes,^20^, ^21^, ^22^ and metabolism of radioligand was found similar between *on* and *off* conditions. The exact molecular mechanism of altered CB1 receptor availability is unknown but may involve changes in receptor protein expression or cellular trafficking (eg, internalization). Also, a true washout of all antiparkinsonian medication would require a longer withdrawal of medication, lasting for days or even weeks, but this was not considered possible for ethical reasons. Thus, the purpose was to investigate subjects with PD in *on* and *off* stages.

Taking these results together, it could be hypothesized that in the *off* stage of PD, CB1 receptors are downregulated because of the high anandamide levels. The antiparkinsonian medication normalizes the anandamide levels, decreasing the downregulation of CB1 receptors closer to normal. Notably, in this study, the withdrawal of the antiparkinsonian medication was a minimum of 12 hours for levodopa and 24 hours for other antiparkinsonian medication, and thus the change in *V*_T_ might reflect receptor availability because of ligand displacement rather than true change in receptor density.

The potentially beneficial role of pharmacological agents targeting the ECS in the management of PD‐related motor symptoms and especially levodopa‐induced dyskinesias has been studied.^34^ Despite the promising results from animal studies,^35^, ^36^, ^37^ clinical trials have yielded conflicting results. This is most likely due to different CBs used, small subject samples,^38^ and the lack of placebo control group.^39^, ^40^ In addition, considering the biphasic dysregulation of CB1 receptors, the effect of ECS targeting agents might vary depending on the stage of the disease. Thus, further placebo‐controlled studies with larger sample sizes and subjects in different stages of PD are required to determine whether CBs could be of benefit in managing the motor symptoms of PD.

Besides the dopaminergic medication, also sex differences in CB1 tracer binding should be taken into account. In our study, in the HC group, [^18^F]FMPEP‐*d*_2_ *V*_T_ was significantly higher in females compared with males in the occipital lobes. These findings differ from a previous study using [^18^F]FMPEP‐*d*_2_, in which a higher *V*_T_ of [^18^F]FMPEP‐*d*_2_ was found in males compared with females.^25^ The inconsistency between the results may be because of differences in age of the participants and hormonal status of female subjects. As their study sample consisted of 11 healthy males (mean age, 27 ± 6 years) and 11 healthy fertile‐age females (mean age, 28 ± 10 years), the HCs in our study were considerably older and postmenopausal women. None of the participants in our study were receiving hormone replacement therapy. The ECS and sex hormones are known to interact bidirectionally, with endocannabinoids downregulating hypothalamic–pituitary–gonadal activity, and gonadal hormones modulating protein expression in the ECS.^41^ More studies are needed to know how the ECS and CB1 receptor availability changes during aging and hormonal changes relate in the general population so that confounding factors can be taken into account more precisely. Similar differences in [^18^F]FMPEP‐*d*_2_ binding between sexes were not observed in the PD group.

In conclusion, this study shows that compared with HCs, subjects with PD have lower CB1 receptor availability in the *off* stage, but binding returns closer to normal levels after medication. Follow‐up studies with larger sample sizes and subjects with various disease durations are needed to further understand the changes in the ECS during the course of PD and the functional interaction between the ECS and the dopaminergic system.

## Author Roles

1. Research project: A. Conception, B. Organization, C. Execution.
2. Statistical Analysis: A. Design, B. Execution, C. Review and Critique.
3. Manuscript Preparation: A. Writing of the first draft, B. Review and Critique.

R.M.A.: 1C, 2A, 2B, 3A, 3B.

H.A.: 1C, 3B.

J.M.T.: 1C, 2B, 3B.

J.E.S.H.: 2C, 3B.

T.V.: 2A, 2C, 3B.

S.L.: 1C, 3B.

J.O.R.: 1A, 1B, 2C, 3B.

A.E.B.: 1A, 1B, 1C, 2C, 3B.

## Financial Disclosures

The authors declare that there are no conflicts of interest relevant to this work.

## Supporting information


**Table S1** The details on the dopaminergic medication for each subject with Parkinson's disease

## Data Availability

The data that support the findings of this study are available on request from the corresponding author. The data are not publicly available due to privacy or ethical restrictions.
