# Supplementary material for: Cannabinoid Receptor Type 1 in Parkinson's Disease: A Positron Emission Tomography Study with [ 18F]FMPEP‐d 2
Source: Mov Disord. 2022 Jun 8;37(8):1673–82. doi: 10.1002/mds.29117 (PMC9544132; doi:10.1002/mds.29117)
Supplement: Supplementary file 1 — Table S1 The details on the dopaminergic medication for each subject with Parkinson's disease [file MDS-37-1673-s001.docx]

**Supplementary Table 1.** The details on the dopaminergic medication for each subject with Parkinson’s disease.

| ID | MAOIs, mg  (LED, mg) | DAs, mg  (LED, mg) | L-dopa, mg  (LED, mg) | LEDD, mg |
| --- | --- | --- | --- | --- |
| C1 | Rasagiline 1 (100) | Pramipexole 2.1 (210) | LC 400/100 (400) | 710 |
| C2 | Selegiline 10 (100) | Pramipexole 0.52 (52) | LCE 500/125/800 (500) | 652 |
| C3 | Selegiline 5 (50) | Pramipexole 1.57 (157) | LCE 300/75/600 (300) | 507 |
| C4 | Selegiline 10 (100) | Pramipexole 1.05 (105) | LC 350/87.5 (350) | 555 |
| C5 | Selegiline 5 (50) | Pramipexole 1.57 (157) | - | 207 |
| C6 | - | Pramipexole 0.26 (26) | LC 200/50 (200) | 226 |
| C7 | - | Ropinorole 8 (160) | LCE 500/125/800 (500) | 660 |
| C8 | Rasagiline 1 (100) | Pramipexole 1.57 (157) | LCE 200/50/800 (200) | 457 |
| C9 | Selegiline 10 (100) | Pramipexole 2.1 (210) | LC 300/75 (300) | 610 |
| C10 | Selegiline 10 (100) | Pramipexole 0.35 (35) | LB 450/112.5 (450) | 585 |
| C11 | Rasagiline 1 (100) | Ropinorole 16 (320) | LC 200/50 (200) | 620 |
| C12 | - | Pramipexole 0.26 (26) | LC 300/75 (300) | 326 |
| C13 | Selegiline 10 (100) | Ropinorole 8 (160) | LB 400/100 (400) | 660 |
| C14 | Rasagiline 1 (100) | Pramipexole 1.57 (157) | LC 750/187.5 (750) | 1007 |
| C15 | Selegiline 10 (100) | Pramipexole 1.57 (157) | LC 200/50 (200) | 457 |
| C16 | Selegiline 5 (50) | - | LC 450/112.5 (450) | 500 |

MAOIs, monoamine oxidase inhibitors. DAs, dopamine agonists. L-dopa, levodopa. LC, levodopa/carbidopa. LCE, levodopa/carbidopa/entacapone. LB, levodopa/benserazide. LED, levodopa equivalent dose. LEDD, levodopa equivalent daily dose.
